# The Book World of Medicine and Science

**Published:** 1898-07-02

**Authors:** 


### The Book World of Medicine and Science.

Transactions of the Royal Academy of Medicine in Ireland. Vol. XV. Edited by John B. Story, M.B., F.R.C.S.I. (Dublin: 1897. Fannin and Co., Limited. Pp. xli., 483, 8vo. With plates and illustrations.)

This handsome, if rather bulky, volume contains the record of a year's work in the important Academy by which it is published. The papers composing it are inevitably of very unequal merit, but the best of them are of more than usual value. The place of honour is deservedly given to Professor Wilhelm His's address on "The Development of the Brain," which is both lucid and well illustrated. Sir William Thomson's well-known address on "Some Surprises and Mistakes" is of great clinical value, both as a personal record and from the wise spirit in which it is written. Dr. MacDowel Cosgrave contributes useful accounts of concurrent scarlatina and enteric fever and of two cases of relapse in scarlatina, and Dr. H. C. Drury a temperate advocacy of the use of guaiacol in pyrexia. The most interesting surgical papers are those on paracentesis pericardii by Mr. Austin Meldon, which evoked a thorough and not wholly favourable discussion; on 61 cases of partial and complete excision of the tongue by Mr. Wheeler; and on a new pattern of decalcified bone ring for intestinal anastomosis by Mr. C. B. Ball. As is only to be expected the contributions of the obstetrical section are numerous and important. We may select for especial mention Dr. F. W. Kidd's cases of ectopic gestation and Dr. Atthill's observations on the anticipation of post partum hæmorrhage. In the other sections the papers most calling for notice are those of Professor Bennett on backward dislocation of the clavicle, illustrated by one case and two dissections, and of Sir Charles Cameron on the recent failure of hospital accommodation in Dublin. The illustrations are throughout excellent.

Burdett's Hospitals and Charities, 1898: Being the Year Book of Philanthropy and the Hospital Annual. By Sir Henry Burdett, K.C.B. (London: The Scientific Press. 1898. Pp. 968. Price 5s.)

This volume of "Burdett's Hospitals and Charities" fully maintains the reputation which has been earned by previous issues. We need not enter into any special details regarding that large portion of the work which is of the nature of a directory of hospitals, dispensaries, poor-law infirmaries, hospitals for infectious diseases, asylums and lunacy boards, convalescent homes, nursing institutions, and various philanthropic agencies, beyond saying that it presents to the inquirer an immense amount of well-arranged information on all these subjects, the collection of which must have entailed upon the compilers a very great amount of labour, dealing as it does not only with institutions of varied character in England, Scotland, and Ireland, but embracing in its purview matter of a similar nature in regard to institutions situated in our numerous colonies, in the United States of America, and "abroad." The information given in regard to the various hospitals is of a very complete and interesting character, and it is a matter of no small interest to find in how many quarters of the globe English institutions have taken root, and among them those peculiar institutions the English hospital and the English nurse. "Burdett's Hospitals and Charities" is, however, something more than a mere directory. Its successive volumes become to some extent a history of philanthropy, from the fact that its introductory chapters form a review of the matters of chief interest relating to the subjects dealt with which have arisen during the year. On this occasion the chapter on "The Effects of the Diamond Jubilee on the Resources of the Voluntary Charities" forms the *pièce de resistance*.

By the help of many calculations an endeavour is made to show that, notwithstanding all the critics, the Prince of Wales's Hospital Fund has really succeeded in doing what it proposed to do, namely, to secure annual subscribers of small sums from among those who previously had not subscribed, and the conclusion is arrived at that, "apart from the givers of the £103,889 included under the head of donations to the Prince's Fund, at least 1,100,000 persons gave something in response to the Prince's letter." This, as Sir Henry Burdett says, "is a surprising result," and we can only hope that he will turn out correct in his estimate of the future of the Fund. Turning aside, however, from questions relating to hospitals, there is a great deal in this book that is instructive and suggestive in regard to charity at large. It is an interesting as well as a useful volume, and its lesson seems to be that charity leads to charity, that giving teaches how to give still more, and that the natural end of a steadily maintained annual subscription is a legacy. The mere business aspect of charity is a far more serious affair than some imagine.

Medical and Surgical Reports of the Boston City Hospital. Ninth Series. Edited by Charles F. Folsom, M.D., W. T. Councilman, M.D., and Herbert L. Burrell, M.D. (Boston, U.S.A. Published by the Trustees. 1898. Pp. 276.)

This volume is much less statistical and in consequence more interesting than might be inferred from its title. The first four articles all refer to diphtheria, of which a large number of cases are treated in the south department of the hospital. The opening paper is a very brief clinical study of 800 cases by Dr. J. H. McCollom. Of these cases 121, or rather over 15 per cent., died. The author establishes that a very rapid pulse with a comparatively low temperature is of the gravest prognostic import. With regard to operations, 79 cases underwent intubation, of which 42 died, two of which had tracheotomy performed secondarily; primary tracheotomy was done twice only, one case recovering. Intubation is in America considered the operation of choice for children. The author concludes also that albuminuria is practically constant in diphtheria, and that the death-rate has been materially reduced by the use of anti-toxin. Subsequent papers discuss the cardiac, nervous and other complications of diphtheria. Among other articles of interest must be noted the collection of statistics relating to major amputations by Dr. J. T. Bottomley. These show that during a little over thirty years the percentage of major amputations to total admissions has fallen from 2 to 0·6 per cent., while at the same time the mortality has declined from 34 to 12 per cent. Eloquent testimony is thus borne to the way in which conservative surgery and antisepsis have advanced side by side. Of the remaining essays we may select for especial notice those on the local anæsthetics used in the eye, by Dr. W. B. Lancaster; on gonorrhœal endocarditis, by Dr. Sears; on hæmatoporphyrinuria, by Dr. Ogden; and on amœbic enteritis, by Dr. L. W. Strong.

